# A centralized decision-making support consultation response for fertility preservation in breast cancer patients: benchmark performance of generative large language models in terms of reliability and readability

**DOI:** 10.3389/fpubh.2026.1845389

**Published:** 2026-05-08

**Authors:** Jincheng Wang, Yang Yang Zhan, Yikuan Shen, Feng Cao, Jiayan Ling

**Affiliations:** Department of Intensive Care Unit, Zhejiang Hospital, Hangzhou, China

**Keywords:** centralized decision-making, fertility preservation, large language models (LLMs), readability, reliability

## Abstract

**Background:**

Large language models (LLMs) hold considerable potential in medical and health education; however, their reliability and interpretability in highly sensitive areas and in decision-making remain unclear. This study focuses on four publicly available LLMs and systematically evaluates their applicability in fertility preservation scenarios for breast cancer patients, thereby providing guidance for targeted use.

**Methods:**

This study utilizes Google Trends to identify and filter information on topics related to fertility preservation for breast cancer patients, and analyses the dialogue outputs of models such as GPT-5.4 Thinking, Gemini 3.0, DeepSeek-V3.2, and Microsoft Copilot. To ensure consistency in responses and the fairness of LLM baseline performance, only one response is generated per query, and no responses are generated repeatedly; all dialogues are submitted to the four large language models using standardized prompts. The study found that 26 fertility-preserving response outputs in breast cancer patients exhibited varying patterns, revealing characteristics relevant to fertility-preserving treatments and decision-making for breast cancer patients. The study utilized reliability assessment tools, including DISCERN, EQIP (Evaluation of Information Quality to Patients), GQS (Global Quality Score) and JAMA (JAMA benchmark criteria), a comprehensive assessment based on six widely used readability metrics [Automated Readability Index (ARI), Coleman–Liau Index (CLI), Flesch–Kincaid Grade Level (FKGL), Gunning Fog Index (GFI), Simple Measure of Gobbledygook (SMOG) with Flesch Reading Ease Score (FRES)].

**Results:**

The findings indicate that there are statistically significant differences in the reliability of various artificial intelligence programmes when it comes to providing highly sensitive, decision-intensive, multidisciplinary medical consultations regarding fertility preservation for breast cancer patients. The average intraclass correlation coefficients for all LLMs ranged from 0.715 to 0.978 (with all *p*-values < 0.001). Microsoft Copilot demonstrates superior performance in terms of information reliability and structural quality, DISCERN [56.5 (49.25, 61)], [EQIP65.0 (51.25, 75.0)], [GQS3.0 (3.0, 4.0)], JAMA [1.0 (1.0, 2.0)], with a higher score than GPT-5.4 Thinking, Gemini 3.0 and DeepSeek-V3.2, the model is capable of providing more reliable information and better decision-making support. The responses generated by all LLMs are too complex for the general public and fail to meet the recommended reading comprehension standards for years 6 to 8; the writing standards of most outputs are equivalent to those of secondary school education, or the reading level required for legal documents.

**Conclusion:**

This study reveals differences in the information provided by various LLMs regarding fertility preservation decisions for breast cancer patients, and recommends selecting a model suited to the specific clinical context; the Microsoft Copilot model demonstrated the best performance. Although LLMs demonstrate a certain degree of reliability when handling complex health enquiries, none have met the readability benchmark recommended for a year 6 reading level. Future research should focus on improving the reliability and readability of health information generated by LLMs to enhance comprehension among a wider audience.

## Introduction

Breast cancer is one of the most common types of cancer among women worldwide (1). According to data from the World Health Organization (2), an estimated 2.3 million women worldwide were diagnosed with breast cancer in 2022, and 670,000 died from the disease. Surgery, radiotherapy and chemotherapy used in cancer treatment can have adverse effects on reproductive health, leading to short-term, long-term or even permanent gonadal toxicity. This places a significant physical burden on patients and can also exacerbate the psychological, family and social challenges they face. A review of relevant studies indicates that the prevalence of reproductive-related anxiety behaviors among adolescent and reproductive-age breast cancer patients ranges from 21.75 to 80% (3), and is closely associated with depression, reduced quality of life and non-adherence to treatment.

With advances in early screening and comprehensive treatment, an increasing number of women of childbearing age and those approaching the menopause are achieving long-term survival. Although the majority of children, women and survivors with cancer (61%−96%) would like to receive more information about the impact of cancer treatment on their fertility at an early stage of their illness (4). However, research indicates that among those who have a clear desire to have children in the future, fewer than 10% of patients seek advice from fertility specialists (5). Consequently, fertility preservation for breast cancer patients has become a key consideration in oncological supportive care, long-term survival management and patient self-determination.

Fertility preservation refers to the use of biomedical methods to maintain a person's reproductive capacity. This is important when fertility is threatened by cancer treatment, such as radiation or chemotherapy, or by other conditions that may affect fertility (6). It primarily involves the use of surgery, medication, or assisted reproductive technologies to help individuals at risk of losing their fertility preserve their ability to have biological children. Currently, fertility preservation is incorporated into cancer care pathways internationally.

The ESMO (European Society for Medical Oncology) guidelines explicitly state that women with early-stage breast cancer who are of childbearing age or premenopausal should be offered counseling on fertility issues and fertility preservation techniques prior to commencing any systemic treatment (7). The 2025 update to the ASCO (American Society of Clinical Oncology) guidelines emphasizes the assessment and counseling of reproductive risks for cancer patients of reproductive age during diagnosis and throughout their treatment and follow-up (8). Where necessary, patients wishing to have children should be referred to a reproductive medicine team at an early stage, and feasible fertility preservation options should be discussed prior to anticancer treatment.

Counseling on fertility preservation for breast cancer patients is complex and often overwhelming for patients and their families. It typically includes a range of issues such as cancer treatment safety, embryo or oocyte freezing, timing of pregnancy, cultural background, and ethical concerns. The large amount of technical information makes it difficult for non-specialists to understand, and the primarily one-way communication in outpatient clinics does not fully address patients' needs.

At present, generative artificial intelligence, including large language models (LLMs), has become a major source of online health information for the general public (9). A cross-sectional study has revealed that 21.5% of respondents have used ChatGPT to obtain health information online, with some members of the public requesting referrals, changing prescriptions, or even adjusting their treatment regimens based on the model's output (10). In 2024, a nationwide survey in Australia revealed that ChatGPT is widely used by the public to seek health advice (11). For breast cancer patients, who face pressure regarding reproductive decisions, LLMs with their capabilities for instant access and interactive dialogue have become an important alternative to traditional outpatient consultations.

It is worth noting that existing research data does not support the use of content generated solely by large language models (LLMs) in health education. A systematic review and meta-analysis indicated that ChatGPT had an overall accuracy rate of 56% when providing information on medical issues (12). However, there was heterogeneity across studies, and key methodological details—such as the dates of the queries, model versions, and session modes—were not adequately reported. Research in the field of oncology indicates that there is insufficient reporting regarding content completeness, transparency of sources, potential risks and readability (13). A study published in JAMA Network Open found that, in studies on LLMs' responses to questions from radiotherapy patients, the health education information generated by the models was significantly more comprehensive and accurate than that in specialist texts (14). At present, existing evaluative studies continue to focus on individual questions and answers in examinations; these single-question formats lack real-world patient scenarios and are subject to information bias.

At present, the output of medical and health education information from LLMs is concentrated on general medical Q&A and applications such as radiotherapy (15). There is a lack of web-based LLMs that are directly accessible to the general public. This study focuses on evaluating the reliability and readability of such systems in highly sensitive, decision-dependent application scenarios, such as health education regarding fertility preservation for breast cancer patients. This study aims to focus on mainstream web-based LLMs, to evaluate the reliability and readability of the generated health education materials on fertility preservation for breast cancer patients, and to determine the quality of the information and its suitability for readers in public health education settings, this provides a basis for patients to access information with the assistance of LLMs, whilst also laying the groundwork for the subsequent development of safer, more explainable health communication strategies regarding fertility preservation for cancer patients.

## Materials and methods

### Source and Resolution of the Issue

This study utilized the MeSH (Medical Subject Headings) thesaurus, the Google Trends platform, and patient-oriented educational materials and guidelines to construct a preliminary pool of fertility preservation issues for breast cancer patients. This was followed by the removal of duplicates, filtering based on thematic relevance and expert review; following a final discussion by the research team, the 26 questions were categorized into five themes, including Diagnosis and Staging, Treatment and Decision-Making, Adverse Reactions and Medication Safety, Prognosis, and Social/Psychological Vulnerability and Behavioral Risk. Aimed at enhancing the comprehensiveness and universality of the research topic.

#### Question source identification

To ensure the accuracy of the search strategy, research was conducted using the MeSH to identify standard medical subject headings and related terms associated with “breast cancer”, “fertility preservation” and “assisted reproductive techniques”, including “female breast cancer”, “breast cancer” and “reproductive techniques, assisted”, in order to define the scope of the research question.

The study further utilized the Google Trends platform to conduct a longitudinal analysis of changes in online search interest for relevant topics over the past 5 years (2020–2025), to identify the key areas of public concern regarding fertility preservation for breast cancer patients worldwide, and to objectively reflect the evolving nature of their health information needs in this field (16). Set the search location to “Worldwide”, the time range to “Last 5 years”, the category to “All categories”, and the search type to “Web Search (Google Web Search)”. The research data was accessed in Hangzhou, Zhejiang, on 18 February 2026. Given that search engine data reflects only the public's online search behavior, there may be a bias in the information. The question bank has been further supplemented and optimized by drawing on relevant guidelines and patient education materials published by authoritative organizations, including the American Society for Reproductive Medicine (ASRM), the American Society of Clinical Oncology (ASCO) and the European Society for Medical Oncology (ESMO).

All preliminary questions were independently reviewed by experts in the relevant fields to eliminate duplicates or content irrelevant to the research objectives. Representatives without a medical background then verified the clarity of the retained questions to ensure they were well worded, easy to understand, and written in language that closely reflects how the general public seeks information.

**Figure 1:**
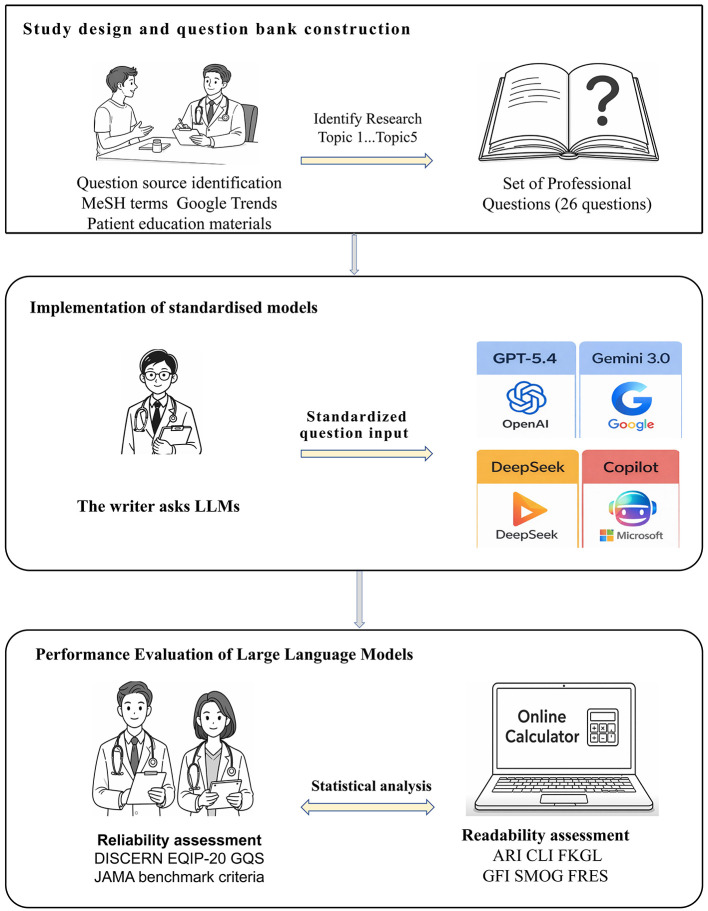
Flowchart of the research process for reference information on the systematic evaluation of decision-making behavior.

Following screening and consolidation, five dimensions and 26 representative clinical questions were ultimately identified for use in assessing the quality, reliability and readability of LLMs responses (See Table 1). The research implementation flowchart is shown in Figure 1.

**Table 1:** Global search queries related to fertility preservation from 2021 to 2025.

Dimensions	Question
Diagnosis and staging	Q1: The presence of occasional breast lumps and discomfort: is it indicative of breast cancer?
	Q2: I have been diagnosed with breast cancer. Could you please provide a professional explanation of what breast cancer entails?
	Q3: How is breast cancer staged?
	Q4: I may have triple-negative or HER2-positive breast cancer. Is this considered a high-risk condition?
	Q5: I have been diagnosed with breast cancer. Can I proceed with my fertility preservation plans as scheduled?
Treatment and decision-making	Q6: What are the treatment modalities for breast cancer, and is a complete cure achievable?
	Q7: Will the treatment modalities for breast cancer impact my fertility?
	Q8: Does radical mastectomy impact pregnancy and lactation?
	Q9: What constitutes fertility preservation?
	Q10: What constitutes an optimal fertility preservation strategy?
	Q11: Is Premature Fertility Preservation a Consideration in Chemotherapy?
	Q12: How should I weigh the timing of fertility preservation against the therapeutic efficacy of breast cancer treatment?
Adverse reactions and medication safety	Q13: What is the recommended time frame for pursuing fertility planning following the completion of breast cancer treatment?
	Q14: What are the potential impacts of chemotherapeutic agents for breast cancer on fertility?
	Q15: Does tamoxifen hormonal therapy have an impact during pregnancy?
	Q16: How does chemotherapy for breast cancer damage my fertility?
	Q17: What is the current status of the development of fertility preservation technology?
	Q18: Can the endocrine changes during pregnancy lead to cancer recurrence?
Prognosis	Q19: I will pay attention to the data report on the survival rate of breast cancer. Can I use it as a reference?
	Q20: How should I correctly understand the risks related to breast cancer treatment and fertility?
	Q21: If my breast cancer has metastasized, can I still preserve my fertility?
	Q22: If I choose fertility preservation, what are the chances of success after breast cancer treatment?
Social/psychological vulnerability and behavioral risk	Q23: After I was diagnosed with breast cancer, I was often anxious about issues related to giving birth and breastfeeding.
	Q24: Decisions made by family, partners, etc. can put me in a predicament regarding fertility preservation.
	Q25: How should I choose a suitable medical institution?
	Q26: How can the different disciplinary backgrounds of breast cancer and fertility provide support for me?

### Model selection

This study evaluated four publicly available web-based large language models (LLMs), the output data for all models was collected in Hangzhou, Zhejiang, on 4 March 2026. The experiment did not employ a subscription-based configuration scheme, nor did it provide additional system role instructions or expert guidance prompts. To ensure consistency in the representation across all models, all queries are entered in a standardized text format. All interactions were carried out using a standard, free personal account; no enterprise licenses, API keys or developer subscriptions were used, in order to accurately reflect the user experience of the general public. The specific model identifiers, access conditions and response configurations are as follows:

GPT-5.4 Thinking: access via the official OpenAI ChatGPT web interface (chat.openai.com), Select the “GPT-5.4 Thinking” version.

Gemini 3.0: access via the Google Gemini web application (gemini.google.com); the interface model version is “Gemini 3.0”.

DeepSeek-V3.2: access via the official DeepSeek platform (chat.deepseek.com) and set the model selection option to “DeepSeek-V3.2”.

Microsoft Copilot: access via the Microsoft Copilot portal (copilot.microsoft.com), with the conversation style set to “Balanced”.

This study has consolidated the 26 questions into a standardized questionnaire. All questions were entered on 4 March 2026 via the publicly accessible web interface of the Lenovo Browser (https://go.lenovo.com.cn/?c=YT_20h2_preload), without using any subscription settings. All LLMs are available as publicly accessible web-based versions.

By default, the models conduct conversations under general public user conditions; no additional system role settings have been configured, nor have any specialized prompts been provided, such as “Please respond as a healthcare professional” or “Please respond as a technical expert”, etc. Aims to objectively reflect the information output by LLMs in real-world usage scenarios where the model has not been fine-tuned, using the default interface preferred by the general public.

Given the specific nature of the model and the lack of transparency regarding internal code, training datasets and so on, to avoid the potential bias in results caused by session history and cached data, disable the memory feature in the LLM settings, clear your browser cache before each query, and start a new session (17). All questions are entered into each model in a standardized text format to ensure consistent wording.

To assess the fundamental stability of the model's output performance, this study selected five questions for each of the included LLMs and conducted preliminary experiments involving independent conversations, repeated three times. By establishing default public user conditions, each LLM exhibits a consistent output structure and style prior to formal questioning, thereby ensuring the consistency of the research.

### Reliability assessment

To systematically evaluate the reliability of health information generated by LLMs for the general public. This study employs four widely used evaluation tools in research on health education materials to conduct a structured assessment of the reliability and quality of information generated by LLMs across various dimensions (18). Notably, DISCERN, EQIP (Evaluation of Information Quality to Patients), GQS (Global Quality Score) and JAMA (JAMA benchmark criteria).

DISCERN: the DISCERN tool was developed in 1999 by the University of Oxford and the National Health Service in the UK to systematically assess the quality of patient-oriented, treatment-related health information, including its reliability, transparency, and decision support (19). In terms of structure, the tool comprises 15 specific items and one overall quality item, representing the quality of treatment-related health information and overall quality, respectively. Each item is scored on a 1–5 Likert scale, with a total score ranging from 16 to 80. A score of 63–80 indicates high quality, 51–62 indicates average quality, 39–50 indicates fair quality, 27–38 indicates poor quality, and 16–26 indicates very poor quality.

EQIP (evaluation of information quality to patients): used to systematically assess the overall production quality of patient education materials, with a focus on whether the information is presented clearly, whether the structure is logical, and whether the content is patient-centered (20). This study utilized the original EQIP-20 instrument to ensure consistency with previous research on health education information. The tool comprises 20 items. For each item, the assessment is rated as “Yes”, “No”, “Uncertain” or “Not applicable”, depending on whether the content meets the criteria. A percentage score is then calculated based on the valid items; a higher score indicates better quality of patient education materials.

GQS (Global Quality Score): suitable for a quick and intuitive overall assessment of the quality of health information; the scoring scale ranges from 1 to 5 (21). 1 indicates very poor quality and a likely tendency to be significantly misleading; 2 indicates poor quality and limited accuracy; 3 indicates average quality, with some information being valid; 4 indicates good quality; and 5 indicates that the information is comprehensive, reliable and of considerable reference value.

JAMA (JAMA benchmark criteria): a framework derived from the Journal of the American Medical Association (JAMA) for assessing the quality of medical information. Specifically, it is used to evaluate the reliability and transparency of online medical information (22). The framework covers four areas: authorship and attribution, sources and references, disclosure of interests and responsibilities, and timeliness. Each criterion met earns 1 point, resulting in a total score of 0 to 4, where a higher score reflects greater transparency and credibility of the source.

All text responses generated by the models were independently scored by two experts in the relevant field. Prior to the evaluation, de-identification is carried out, including the removal of interface identifiers, model watermarks and prompt features, the LLMs are then evaluated in a randomized order to minimize evaluator bias. Where there is a discrepancy between the scores given by two independent assessors, the first step is for the two parties to discuss the matter and reach a consensus, if no consensus can be reached following consultation, a third senior specialist with extensive clinical experience shall be called upon to make a final decision.

To quantify the consistency among assessors, the study conducted a statistical analysis of the initial independent scores using the intraclass correlation coefficient (ICC).

### Readability assessment

To assess the difficulty of reading comprehension in the textual responses generated by LLMs. This study utilized an online readability calculation tool (https://readabilityformulas.com) to conduct a quantitative analysis of the model's output (see Table 2 for the calculation formula) (23). Included Automated Readability Index (ARI), Coleman–Liau Index (CLI), Flesch–Kincaid Grade Level (FKGL), Gunning Fog Index (GFI), Simple Measure of Gobbledygook (SMOG) with Flesch Reading Ease Score (FRES).

**Table 2:** Formula for calculating the readability score of an online calculator.

Index	Full name	Formula
ARI	Automated Readability Index	ARI = 4.71 × (characters/words) + 0.5 × (words / sentences) – 21.43
CLI	Coleman–Liau Index	CLI = 0.0588L – 0.296S – 15.8
FKGL	Flesch–Kincaid grade level	FKGL = 0.39 × ASL + 11.8 × ASW – 15.59
GFI	Gunning Fog Index	GFI = 0.4 × [(words/sentences) + 100 × (complex words / words)]
SMOG	Simple measure of gobbledygook	SMOG = 1.0430 × √[30 × (polysyllables/sentences)] + 3.1291
FRES	Flesch Reading Ease Score	FRES = 206.835 – 1.015 × ASL – 84.6 × ASW

Characters, number of letters/numbers; Words, number of words; Sentences, number of sentences; L, average number of letters per 100 words; S, average number of sentences per 100 words; ASL, average sentence length (words / sentences); ASW, average syllables per word (syllables/words); Complex words, words with three or more syllables; Polysyllables, words with three or more syllables; ASL, average sentence length; ASW, average syllables per word.

The ARI, CLI, FKGL, FRES, and SMOG indices reflect the approximate educational grade level required to comprehend textual information (24). Higher scores indicate a higher required reading proficiency level. The FRES uses a scale of 0–100, with higher scores indicating that the text is easier to read. According to recommendations from the American Medical Association (AMA) and the National Institutes of Health (NIH), the reading level required to understand health education materials corresponds to that of pupils in years 6 to 8 in US schools. Furthermore []. If the FRES score is below 80 and the readability scores for the remaining indicators exceed 6, the response text does not meet the recommended readability standards for public health education materials.

### Statistical analysis

This study evaluates the baseline performance of LLMs in delivering health education information on fertility preservation for breast cancer patients across multiple dimensions (25), including DISCERN, EQIP, GQS, JAMA and text readability metrics. Following tests for normality of each evaluation indicator, non-parametric tests were employed; descriptive statistics were presented as medians and interquartile ranges (IQRs).

The Kruskal–Wallis test was used to assess overall differences between the models. When overall differences proved significant, Dunn's *post-hoc* pairwise comparisons were performed, and corrections for multiple comparisons were applied. Consistency between the two evaluations was then assessed using the intra-class correlation coefficient (ICC). All statistical tests were two-tailed, with a significance level set at *P* < 0.05. Statistical analyses and visualizations were conducted using R software (version 4.5.1).

## Results

### Reliability assessment

This study utilized four assessment tools—DISCERN, EQIP, JAMA and GQS—to conduct a multidimensional evaluation of the fertility preservation health education information provided by the included LLMs for breast cancer patients. There were statistically significant differences among the four LLMs in their scores on DISCERN, EQIP, JAMA, and GQS (see Table 3). The average ICC for all LLMs ranged from 0.715 to 0.978 (*P* < 0.001). The results of the Kruskal–Wallis test showed the following for DISCERN (*H* = 31.985, *P* < 0.001), EQIP (*H* = 21.576, *P* < 0.001), JAMA (*H* = 85.055, *P* < 0.001) and GQS (*H* = 15.802, *P* = 0.001).

**Table 3:** Reliability scores across LLMs [median (Q1, Q3)].

	DISCERN	EQIP	GQS	JAMA
DeepSeek-V3.2	36.5 (29.25, 44.75)	40.0 (35.0, 55.0)	3.0 (2.25, 4.0)	0.0 (0.0, 0.0)
GPT-5.4	54.0 (47.75, 60)	60.0 (50.0, 65.0)	4.0 (3.25, 4.0)	0.0 (0.0, 0.0)
Gmini 3.0	55.5 (46.25, 60.75)	60.0 (47.5, 65.0)	4.0 (3.0, 4.0)	0.0 (0.0, 0.0)
Microsoft Copilot	56.5 (49.25, 61)	65.0 (51.25, 75.0)	3.0 (3.0, 4.0)	1.0 (1.0, 2.0)
*H*	31.928	21.576	15.802	85.055
*P*	< 0.001	< 0.001	0.001	< 0.001

Values are presented as median (Q1, Q3), where Q1 and Q3 represent the 25th and 75th percentiles, respectively.

The corresponding H and P values are reported.

**Figure 2:**
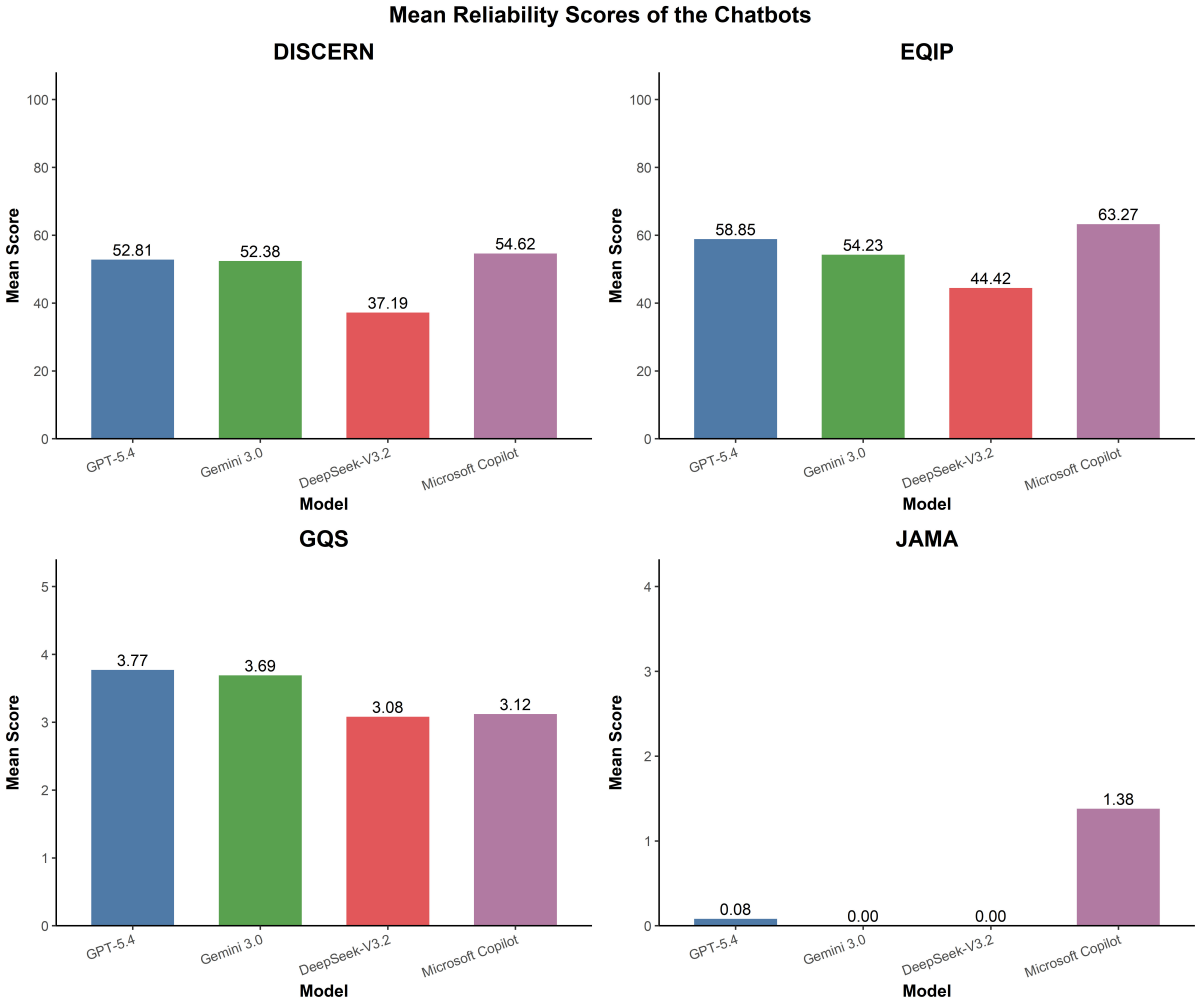
LLM reliability scoring metric.

In the DISCERN score (see Figure 2), DeepSeek-V3.2 has the lowest median (36.5 [29.25, 44.75]), while Microsoft Copilot has the highest median score (56.5 [49.25, 61]). The median values for Gemini 3.0 and GPT-5.4 Thinking are 55.5 [46.25, 60.75] and 54.0 [47.75, 60], respectively, indicating that these two models are of a similar standard overall. Similarly, the EQIP evaluation results show a consistent pattern: DeepSeek-V3.2 again has the lowest median (40.0 [35.0, 55.0]), and Microsoft Copilot records the highest median score (65.0 [51.25, 75.0]). The median for both GPT-5.4 Thinking and Gemini 3.0 is 60, indicating that both groups were at a similar level in the quality of the health education information structure regarding fertility preservation for breast cancer patients.

The GQS score (see Figure 2) shows that the median for both GPT-5.4 Thinking and Gemini 3.0 is 4.0, it is noted that the output receives high marks in terms of structure and readability, based on subjective assessments. The median for both DeepSeek-V3.2 and Microsoft Copilot is 3.0. However, the interquartile range differs between the two, and the subjective quality rating is slightly lower.

In the JAMA score (see Figure 2), the most significant statistical difference was observed across the models (*H* = 85.055, *P* < 0.001). Among these, the median for DeepSeek-V3.2, GPT-5.4 Thinking and Gemini 3.0 is [0 (0, 0)], whilst the median for Microsoft Copilot is [1.0 (1.0, 2.0)]. These results suggest that, by default, the information displayed on the public interface of all models included in the study generally exhibits characteristics of transparency, these include the absence of author details and attribution, sources and references, disclosure of interests and responsibilities, and timeliness.

Further analysis of pairwise comparison results revealed that differences among the four models across various reliability and quality assessment tools indicate significant feature dimension dependence in model performance (see Table 4). DeepSeek-V3.2, GPT-5.4 Thinking, Gemini 3.0 and Microsoft Copilot all exhibit significant differences in the DISCERN and EQIP benchmarks. The GQS results show that DeepSeek-V3.2 differs significantly from both GPT-5.4 Thinking and Gemini 3.0. The difference with Microsoft Copilot is not significant. Distinctions among Microsoft Copilot, GPT-5.4 Thinking, and Gemini 3.0 appear mainly in GQS and JAMA. There are no statistically significant differences between GPT-5.4 Thinking and Gemini 3.0 across the four evaluation dimensions, suggesting broadly comparable reliability and quality for both models.

**Table 4:** Test results of Dunn given in the form of reliability scores (*p*-values).

Comparison	DISCERN	EQIP	GQS	JAMA
DeepSeek-V3.2-Gmini 3.0	0.000	0.022	0.015	1.000
DeepSeek-V3.2-GPT-5.4	0.000	0.001	0.012	0.865
Gmini 3.0 - GPT-5.4	0.980	0.356	0.909	1.000
DeepSeek-V3.2-Microsoft Copilot	0.000	0.000	0.893	0.000
Gmini 3.0-Microsoft Copilot	0.757	0.108	0.013	0.000
GPT-5.4-Microsoft Copilot	0.919	0.450	0.015	0.000

### Readability assessment

**Figure 3:**
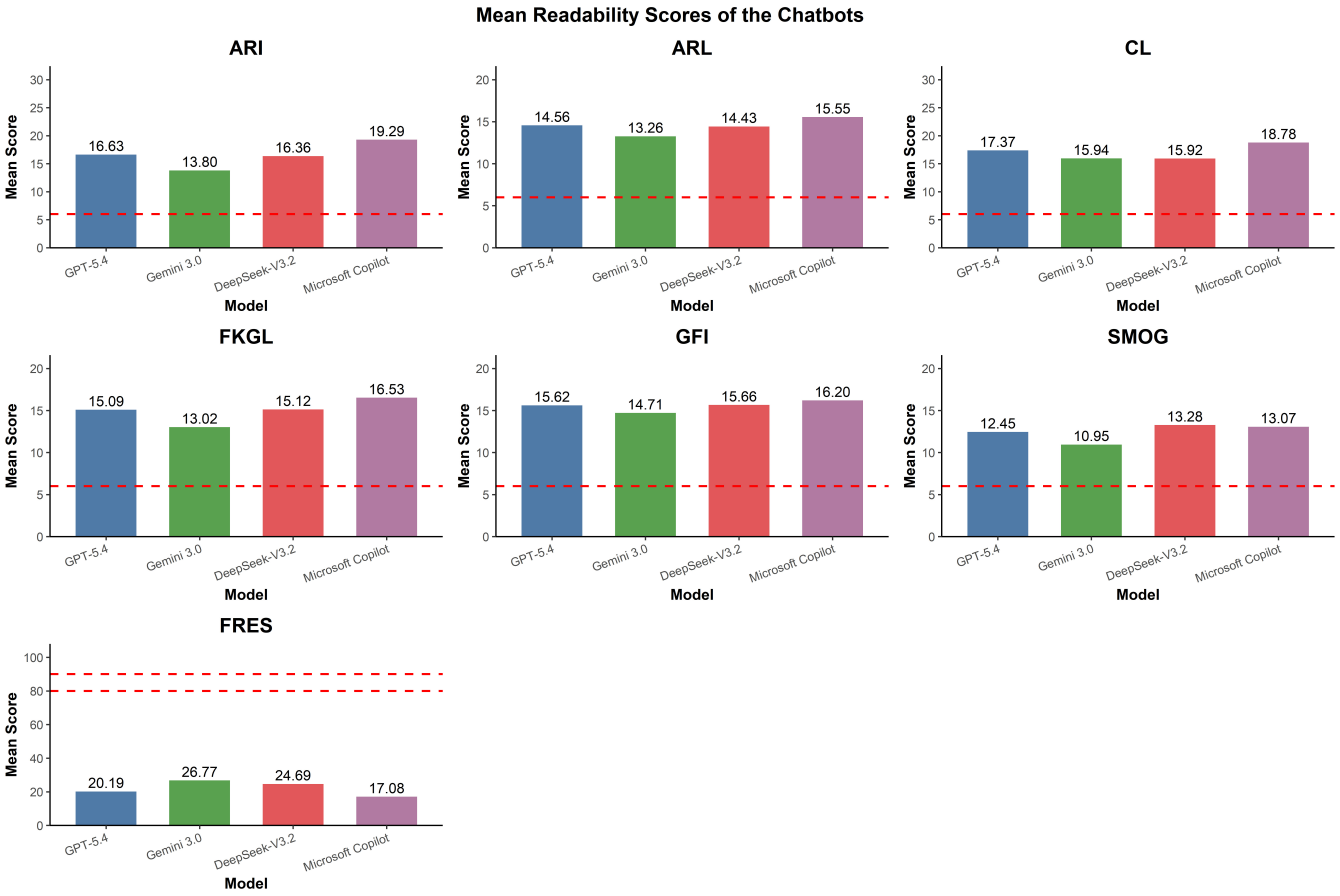
LLM readability scoring indicator.

This study employed six validated readability metrics—ARI, Flesch Reading Ease, Gunning-Fog, FKGL, CLI, and SMOG—to assess the readability of health education information on fertility preservation for breast cancer patients generated by four LLMs (see Table 5). The results indicate that, for ARI, GFI, FKGL, CLI, and SMOG, the median scores of LLM responses were significantly higher than the reading comprehension levels of year 6–8 pupils (aged 11–12) for general public health education materials. This suggests that these LLM responses generally require a higher reading level. As shown in the figure (see Figure 3), Gemini 3.0 has a relatively low prompt reading load, with median ARI, FKGL, GFI and SMOG values of 13.1 (10.7, 16.5), 12.6 (10.6, 14.7), 14.4 (13.2, 15.8) and 10.0 (8.8, 12.9), whilst the median FRES value was the highest at 27.0 (15.8, 34.8). However, it remains significantly below the recommended readability range. Microsoft Copilot achieved the lowest median FRES score [13.0 (4.0, 20.8)], whilst its scores for all other metrics were relatively high, suggesting that its output text is highly complex and difficult to read. DeepSeek-V3.2 and ChatGPT-5.4 exhibit readability metrics that fall between the two aforementioned models, yet neither has achieved the ideal level for public dissemination.

**Table 5:** Readability scores across LLMs [median (Q1, Q3)].

Program	ARI	CLI	FKGL	FRES	GFI	SMOG
DeepSeek-V3.2	16.9 (13.4, 18.2)	15.9 (15.2, 17.2)	15.0 (12.4, 17.0)	22.5 (13.5, 33.8)	16.1 (14.5, 17.3)	13.1 (11.4, 14.7)
GPT-5.4	16.1 (12.8, 18.6)	17.9 (15.1, 19.7)	15.2 (11.8, 16.6)	15.0 (4.3, 31.8)	16.0 (13.7, 17.3)	12.2 (10.5, 13.5)
Gmini 3.0	13.1 (10.7, 16.5)	15.8 (13.7, 17.6)	12.6 (10.6, 14.7)	27.0 (15.8, 34.8)	14.4 (13.2, 15.8)	10.0 (8.8, 12.9)
Microsoft Copilot	17.9 (14.4, 21.8)	18.7 (16.1, 19.7)	16.4 (13.7, 18.1)	13.0 (4.0, 20.8)	16.2 (14.8, 17.5)	12.9 (11.1, 15.1)

Values are presented as median (Q1, Q3), where Q1 and Q3 represent the 25th and 75th percentiles, respectively.

Overall, none of the LLM-provided health education information on fertility preservation for breast cancer patients met the readability standards recommended for the general public, potentially placing a significant burden on reading and comprehension for groups with lower e-health literacy (26).

## Discussion

This study compared the performance of four mainstream web-based LLMs, accessible to the general public, in delivering health education information on fertility preservation for breast cancer patients. The results showed that there were significant differences (*P* < 0.001) among the four LLMs across the four reliability metrics DISCERN, EQIP, GQS and JAMA. This difference is reflected in the core assessment tools in terms of the quality of the information's structure, its transparency and the clarity of its presentation. At the same time, none of the LLMs met the recommended standards for health education materials across all six readability metrics.

This finding suggests that, when dealing with highly specialized health issues relating to clinical decision support, improvements in information quality, transparency of sources and accessibility do not necessarily occur simultaneously.

### Imbalance between reliability and readability

This study found that, in the information provided by LLMs in response to queries regarding fertility preservation for breast cancer patients, there is a certain imbalance between the completeness of the information structure and the accessibility of the language. Microsoft Copilot performed best in the DISCERN and EQIP scores, suggesting that its content is relatively comprehensive in terms of organization, treatment information and patient education, however, it achieved the highest grade-level score and the lowest FRES score, this indicates that the text of the model is of a professional standard, which may hinder the general public's ability to understand it effectively. Gemini 3.0 performs best on readability metrics, but the output is difficult for patients to read. A systematic review has shown that LLMs have limited capacity to generate patient health education materials that meet optimal readability standards, exhibiting structural differences arising from the reliability-readability trade-off and a dependence on the architecture of the LLMs (27). Research in the field of public health by Qiu et al. (28) reveals a significant disparity between the reliability of model inference and the readability of effective communication.

The findings of the JAMA evaluation have certain clinical implications, with the exception of Microsoft Copilot, the other models lack author attribution, references, disclosures of conflicts of interest and information regarding timeliness. For breast cancer patients with limited time who are facing important decisions regarding fertility, a lack of transparency can lead to potential trust issues. The general public often reduces reliance on authoritative information and specialized consultations due to the fluidity and completeness of information structures (29). Previous research on cancer has shown that health education information generated by LLMs is appealing in terms of empathy and readability. This study further demonstrates that robust evidence and traceability are areas where LLMs fall short when generating health education content (30).

### The characteristics of LLMs determine the differences in model outputs

Differences in architecture and parameters between various LLM systems influence the content characteristics of the health education materials they generate. MI-CLEAR-LLM 2025 further emphasizes that model identification, access methods, prompt execution, and randomness control all influence the output (31). Consequently, the differences observed in this study represent a cross-sectional snapshot of the performance of web-based LLMs under specific testing conditions, rather than a definitive conclusion regarding their underlying mechanisms. The strengths of DeepSeek-V3.2 in subjective quality assessment stem from the model's ability to perform in-depth reasoning (32), whilst striving for logical and informational completeness, it excels at generating lengthy text and complex sentence structures. However, it must be acknowledged that this self-contained logic lacks key elements necessary to support decision-making. In their study on patient education materials in dentistry, Arunachalam et al. found that ChatGPT-4.0 demonstrated superior comprehension, whilst there were significant differences between models in terms of readability, usability and comprehensibility (33). In their study on patient education for coeliac disease, Cao et al. similarly found that the content generated by all models had an error rate of 13.3%−24.2% (34). Consequently, when taken together with the findings of this study, this further indicates that, it is difficult for the output of a single model to achieve optimal performance across all evaluation metrics, and the quality of output from different large language models (LLMs) is significantly dependent on the specific task at hand.

### Readability and cognitive load

The findings of this study indicate that the quality of information has not translated into greater accessibility for the general public. Relevant reviews indicate that most LLMs are unable to generate standardized information in the field of public health that is suitable for recommendation (35). Gemini 3.0 is the closest of the four models to the recommended reading level, but its results are still significantly higher than the year 6 level recommended by the AMA and the year 8 level recommended by the NIH. This suggests that even when a model appears clear and reliable in terms of content organization, its output is still difficult to adapt directly for the general public to read. A systematic review and meta-analysis of online patient education materials for breast cancer found that the average readability level was approximately 11.81 years, nearly twice the upper limit recommended by the AMA (36). Similarly, research into cancer-related chatbots on topics such as chemotherapy-induced cardiotoxicity suggests that the quality of content can reach acceptable or even high levels, though the average reading level remains around year 13 (37).

There appears to be a potential inverse correlation between the reliability and readability of health education materials (38). In this study, Microsoft Copilot's superior performance on reliability metrics was often accompanied by a decline in readability. In their study on information extraction from electronic health records in older adults care. Alkhalaf et al. noted that there is a trade-off between the performance of LLMs across multiple dimensions, including accuracy, robustness, fairness and relevance; optimizing a single dimension may come at the expense of others, highlighting the model's dependence on specific features (39). In their study on educational materials for patients with inflammatory bowel disease, Gatiganti et al. found that even when prompts specifically instructed the models to explain concepts at a year 6 reading level, the outputs generated by five mainstream LLMs still failed to consistently meet the recommended standards (40). When the complexity of the text remains at the level of secondary school or even university reading, the information's accessibility decreases significantly, thereby undermining its effectiveness in patient education.

### Highly sensitive and decision-support-oriented

The fertility preservation health education consultation for breast cancer patients in this study differs from the acquisition of general health education information. Fertility preservation in breast cancer patients involves strict time requirements and interdisciplinary integration (41). For women of childbearing age with breast cancer, controlled ovarian stimulation and egg retrieval must be carried out within a strict time window (10–12 days) (42). Timely referral and clear communication are, therefore, crucial to clinical decision-making. Both ASCO and ESMO emphasize that discussions regarding fertility risks should be concluded before commencing anticancer treatment (43). Questions of this nature require not only an answer to the question of “whether fertility can be preserved”, but also a clear explanation of the choice of treatment options, decisions regarding the fertility preservation pathway and timing, and information on alternative options.

The performance of DeepSeek-V3.2 on the DISCERN and EQIP scales indicates that the model fails to disclose the risks and benefits of fertility preservation options, thereby affecting patients' ability to make informed decisions. Research on the HEAL-Summ framework indicates that, when it comes to sensitive health topics (such as mental health, cancer and addiction), particular attention must be paid to the emotional and ethical reliability of health information generated by LLMs, to ensure that their output is both accurate and empathetic (44).

## Limitations

The findings of this study reflect the baseline performance of publicly available web-based LLMs in terms of the quality and reliability of their responses to health education queries on fertility preservation for breast cancer patients, as well as the transparency of their sources and readability, as of 4 March 2026. They do not represent the stable output of subsequent model updates and iterations, and therefore have certain limitations. Currently, the public needs to exercise caution when receiving highly specialized health education information targeting topics such as fertility preservation for breast cancer patients (45).

LLMs are constantly evolving, and their performance characteristics change as the models are updated (46). Current evaluations of large language models in healthcare settings do not adequately account for real patient data, and readability metrics merely reflect linguistic complexity and fail to capture the genuine, subjective experiences of the general public (47). Current research is limited to the textual level and has not yet been validated to determine whether patients truly understand the information, whether this improves their preparation for medical appointments, or whether it influences the quality of their reproductive decisions.

Further studies should consider conducting repeated sampling and longitudinal research to assess the stability of web-based outputs. At the same time, we will assess comprehension, decision-making conflict, empathy and trust among real patients, thereby determining the practical limitations of the model in fertility preservation education for breast cancer patients. As LLMs undergo rapid iteration, a continuous monitoring framework should be established to balance reliability and interpretability (48).

## Conclusion

In summary, this study is based on a single major version of LLMs deployed at a specific point in time and in their publicly available form. The results indicate that, in the context of fertility preservation counseling for breast cancer patients, the responses provided by the current model exhibit certain limitations in striking a balance between information quality and readability. At present, health education content generated by models based on specific criteria must undergo professional review and linguistic optimization before it is made available to the general public.

In the future, as the model undergoes iterations and its feature architecture is refined, strategies such as optimizing prompts and retrieval-enhanced generation will be employed to enhance the transparency of health education information sources, whilst further improving language quality, emotional tone and readability.

## Data Availability

The original contributions presented in the study are included in the article/supplementary material, further inquiries can be directed to the corresponding authors.
